# Regulation of tight junction assembly and epithelial morphogenesis by the heat shock protein Apg-2

**DOI:** 10.1186/1471-2121-8-49

**Published:** 2007-11-20

**Authors:** Saima Aijaz, Elena Sanchez-Heras, Maria S Balda, Karl Matter

**Affiliations:** 1 Division of Cell Biology, UCL Institute of Ophthalmology, University College London, Bath Street, London, EC1V 9EL, UK

## Abstract

**Background:**

Tight junctions are required for epithelial barrier formation and participate in the regulation of signalling mechanisms that control proliferation and differentiation. ZO-1 is a tight junction-associated adaptor protein that regulates gene expression, junction assembly and epithelial morphogenesis. We have previously demonstrated that the heat shock protein Apg-2 binds ZO-1 and thereby regulates its role in cell proliferation. Here, we addressed the question whether Apg-2 is also important for junction formation and epithelial morphogenesis.

**Results:**

We demonstrate that depletion of Apg-2 by RNAi in MDCK cells did not prevent formation of functional tight junctions. Similar to ZO-1, however, reduced expression of Apg-2 retarded *de novo *junction assembly if analysed in a Ca-switch model. Formation of functional junctions, as monitored by measuring transepithelial electrical resistance, and recruitment of tight and adherens junction markers were retarded. If cultured in three dimensional extracellular matrix gels, Apg-2 depleted cells, as previously shown for ZO-1 depleted cells, did not form hollow polarised cysts but poorly organised, irregular structures.

**Conclusion:**

Our data indicate that Apg-2 regulates junction assembly and is required for normal epithelial morphogenesis in a three-dimensional culture system, suggesting that Apg-2 is an important regulator of epithelial differentiation. As the observed phenotypes are similar to those previously described for ZO-1 depleted cells and depletion of Apg-2 retards junctional recruitment of ZO-1, regulation of ZO-1 is likely to be an important functional role for Apg-2 during epithelial differentiation.

## Background

Epithelial tight junctions are the most apical component of the junctional complex and are critical for epithelial barrier function as they form the paracellular diffusion barrier [1-3]. Tight junctions are composed of several transmembrane proteins that are linked to a cytoplasmic plaque and the actin cytoskeleton [4,5]. This cytoplasmic plaque consists of a protein network formed by adaptor proteins with multiple protein/protein interaction motifs, cytoskeletal linkers and signalling proteins such as protein kinases and phosphatases [6,7]. These junction associated protein complexes also interact with dual localisation proteins that localise to both the junction and the nucleus [8]. Several of these junctional components have been linked to the regulation of epithelial proliferation, differentiation and polarisation [9,10].

ZO-1 is the first tight junction protein identified and functions as a junctional adaptor that interacts with multiple transmembrane proteins, components of the junctional plaque and actin filaments [4,5,11,12]. ZO-1 is expressed by most cells and, in the absence of tight junctions, can associate with other cell-cell adhesion complexes, such as adherens and gap junctions [13-15]. Repression of ZO-1 expression in different epithelial cell lines revealed that ZO-1 is not required for junction formation and polarisation in two-dimensional (2-D) culture systems [16,17]. In three-dimensional cultures (3-D), however, normal ZO-1 expression is required for the formation of polarised hollow cysts, indicating that it plays a role in the regulation of epithelial morphogenesis [18].

ZO-1 has been directly associated with a signalling function of tight junctions. ZO-1 binds with its SH3 domain to the Y-box transcription factor ZONAB (DbpA), which results in cytoplasmic sequestration and inhibition of the transcriptional activity of the latter protein [19]. The ZO-1/ZONAB pathway regulates epithelial proliferation and expression of genes important for epithelial differentiation and cell cycle progression such as erbB-2, cyclin D1 and PCNA [18-20]. The SH3 domain of ZO-1 also interacts with the heat shock protein Apg-2 [21]. Apg-2 and ZONAB compete for binding to ZO-1, resulting in ZONAB dissociation and activation if the interaction with Apg-2 is favoured by conditions such as, for example, heat shock. Expression of all three proteins can be deregulated in different epithelial cancers, suggesting that they might be functionally relevant for the maintenance of the epithelial cell type and tumorigenesis [22-30].

Given the modulatory role of ZO-1 during junction formation, its involvement in large protein complexes, and the role of heat shock proteins as folding and assembly factors, we tested whether Apg-2 regulates junction formation. Our data indicate that Apg-2 is not essential for the formation of functional tight junctions but regulates junction assembly in 2-D cultures similar to its interaction partner ZO-1. In 3-D cultures, however, Apg-2 was required for normal epithelial morphogenesis, suggesting that the heat shock protein regulates pathways important for epithelial polarisation and differentiation.

## Results

### Apg-2 regulates the assembly of functional tight junctions

Apg-2 binds to the SH3 domain of ZO-1, and this domain is important for the regulation of junction assembly in MDCK cells [16,21]. To test whether Apg-2 is also required for junction formation, we made use of previously described MDCK cell lines permitting the conditional depletion of either Apg-2 or ZO-1 [18,21]. In these cell lines, RNA interference is induced by the addition of tetracycline, which inactivates a co-transfected repressor, resulting in expression of shRNAs. We made use of four different cell lines: two expressing two different shRNA constructs that target different sequences of Apg-2 (z2 and z5; [21]), one for the repression of ZO-1 [18], and a control cell line expressing a non-targeting construct.

**Figure 1:**
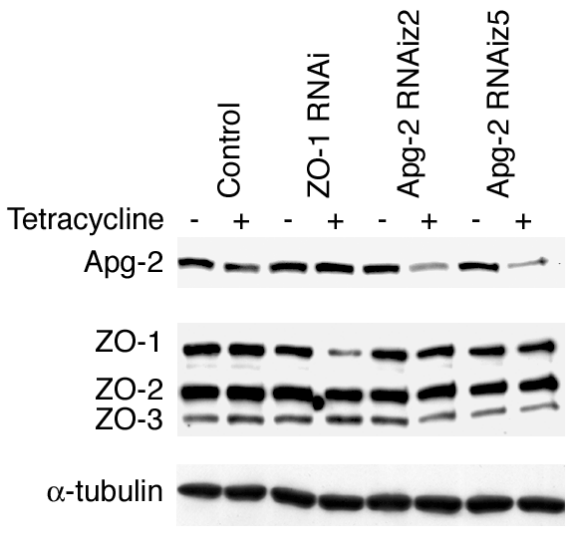
**Repression of Apg-2 and ZO-1 by regulated RNA interference**. Stable MDCK cell lines expressing the tetracycline repressor as well as shRNAs targeting ZO-1 or Apg-2 (z2 and z5 target different sequences), or a non-targeting shRNA under the control of a tetracycline-regulated promoter were plated in low calcium medium onto permeable culture inserts in the presence or absence of tetracycline. After 18 hours, the cells were switched to normal calcium medium for 24 hours adding tetracycline as indicated. The cells were then lysed in SDS-PAGE sample buffer and analysed by immunoblotting. Shown are immunoblots for Apg-2, ZO-1, ZO-2, ZO-3 and α-tubulin. Expression of ZO-1 was reduced to 24 +/- 7% and of Apg-2 to 22 +/- 6% of the respective levels in control cultures (n = 4).

To determine possible effects on junction formation, we combined these cell lines with a calcium-switch protocol, permitting us to study synchronised *de novo *junction formation [31]. To do this, cells were plated at high density on permeable supports, which facilitate the functional analysis of tight junctions [32], in medium containing a low calcium concentration, which does not allow the formation of intercellular junctions. 24 hours later, junction formation was stimulated by changing the medium to standard culture medium. RNA interference was induced by adding tetracycline at the time of plating. Figure 1 confirms that tetracycline induced depletion of ZO-1 and Apg-2 in the respective cell lines, but not in control cells. Depletion of Apg-2 did not significantly affect the expression of ZO-1 or of the interacting proteins ZO-2 and ZO-3 (Fig. 1). Similarly, no differences in the expression of occludin and claudin-4 were observed (not shown).

**Figure 2:**
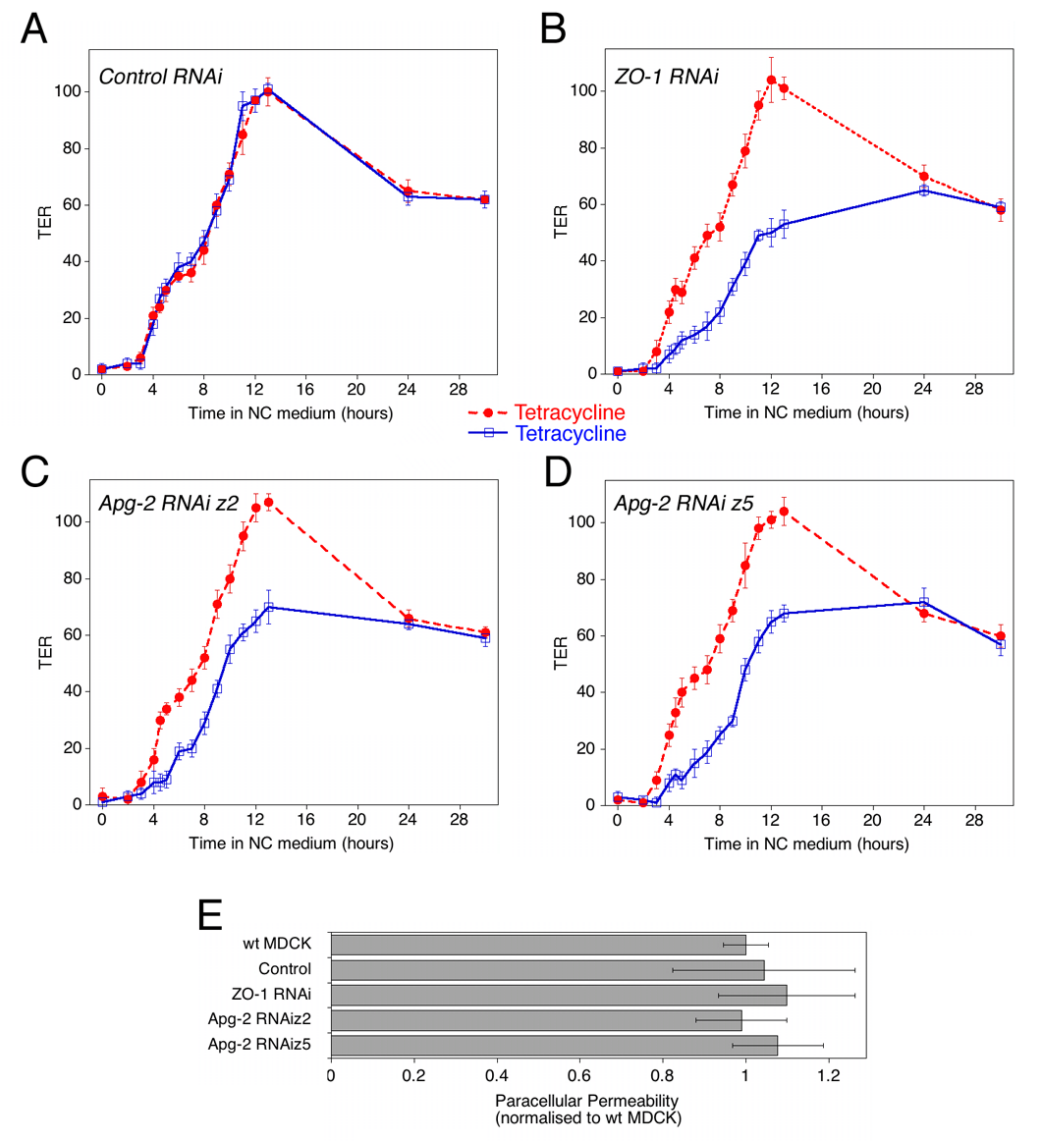
**Development of functional tight junctions**. (A-D) Cells were plated and incubated without or with tetracycline as described in figure 1. After switching from low calcium to normal calcium medium, TER was measured at the indicated time points. Shown is a typical experiment using a control RNAi cell line (A), a ZO-1 RNAi cell line (B), and two Apg-2 RNAi cell lines (C and D). Values represent means +/- 1 SD of quadruplicates. (E) Paracellular permeability of fluorescent dextran was measured in the same cultures as those shown in panels A to D 3 days after the initiation of junction formation. Shown are the values obtained with 4 kD FITC dextran with tetracycline-treated cultures. Values represent means +/- 1 SD of quadruplicates. Diffusion of 70 kD dextran was also not affected by depletion of Apg-2 and ZO-1 (not shown).

We next monitored the assembly of functional tight junctions by measuring transepithelial electrical resistance (TER) at different times after adding calcium. Figure 2A shows that tetracycline did not affect the assembly of tight junctions: with and without the antibiotic, cultures reached maximal TER within about 12 hours (maximal TER values were between 200 and 240 Ωcm^2^). TER then decreased again to about 60% of maximal values after 24 hours. After 2 days, cells reached steady state values of about 60 Ωcm^2^. This is the typical profile of TER development that has previously been described for MDCK strain II cells [31].

Figure 2B shows that depletion of ZO-1 resulted in the previously described retardation of junction formation [16,17]. TER developed more slowly and only reached 50% of the value of control cultures after 12 hours. After 24 hours, however, the values of depleted and non-depleted cultures were the same. If Apg-2 was depleted, TER development was also retarded and cultures reached about 60% of the value of control cultures after 12 hours (Fig. 2C and 2D). As in cells with reduced ZO-1 expression, the TER values measured in depleted and non-depleted cultures were the same after 24 hours. Similarly, all cell lines reached steady state values of about 60 Ωcm^2 ^after 2 days. Thus, normal expression of Apg-2, like ZO-1, is not required for the formation of electrically resistant tight junctions, but the heat shock protein regulates the kinetics of junction formation. Normal expression levels of Apg-2 seem to be sufficient to support efficient junction assembly, as overexpression did not affect the development of TER in a calcium switch (not shown).

Tight junctions restrict paracellular permeability of ions as well as of hydrophilic molecules based on their size. As the two parameters are not always regulated in the same way, we measured paracellular permeability of fluorescent dextrans in steady state cultures. Cultures were incubated for 3 hours with 4 kD or 70 kD fluorescent dextran and diffusion in the apical-to-basolateral direction was then determined by measuring fluorescence in the basolateral medium. Figure 2E shows that neither depletion of ZO-1 nor Apg-2 affected paracellular permeability of 4 kD dextran. We could also not detect an effect on 70 kD dextran diffusion (not shown). Thus, Apg-2- and ZO-1-depleted cells assembled junctions that represented functional paracellular diffusion barriers.

### Depletion of Apg-2 retards the recruitment of ZO-1 and interacting proteins

**Figure 3:**
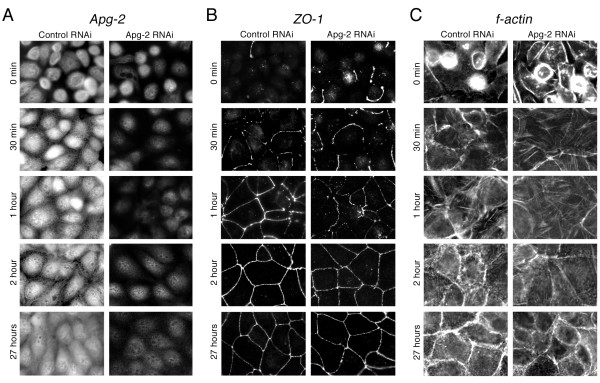
**Retardation of ZO-1 recruitment to forming junctions by Apg-2 depletion**. Control and Apg-2 RNAi cell lines were cultured in low calcium with tetracycline and junction formation was then induced for the indicated periods of time before fixation. The cells were then labelled with antibodies against Apg-2 (A), ZO-1 (B), or fluorescent phalloidin (C). If tetracycline was not added and Apg-2 was not depleted, Apg-2 RNAi cell lines formed junctions at the same speed as control RNAi cell lines (not shown). Note, recruitment of ZO-1 to the forming junctional complex was retarded, but the distribution of ZO-1 appeared to be normal at later time points.

We next tested whether inhibition of TER development reflected a retardation of junction formation that was detectable by immunofluorescence. Hence, we fixed cells after different periods of time with calcium and then monitored the distribution of Apg-2 and junctional markers by immunofluorescence. Figure 3A shows that Apg-2 was present in the nucleus as well as the cytoplasm of control cells at all times. As described previously, weak Apg-2 staining was also observed along the lateral membrane [21]. This appeared to be more evident during early time points (e.g., 1 and 2 hours). However, this does not appear to reflect increased association with ZO-1, as we could not detect preferential co-immunoprecipitation during junction formation as opposed to stable monolayers (not shown). In depleted cells, expression was low as expected from the immunoblots in figure 1.

**Figure 4:**
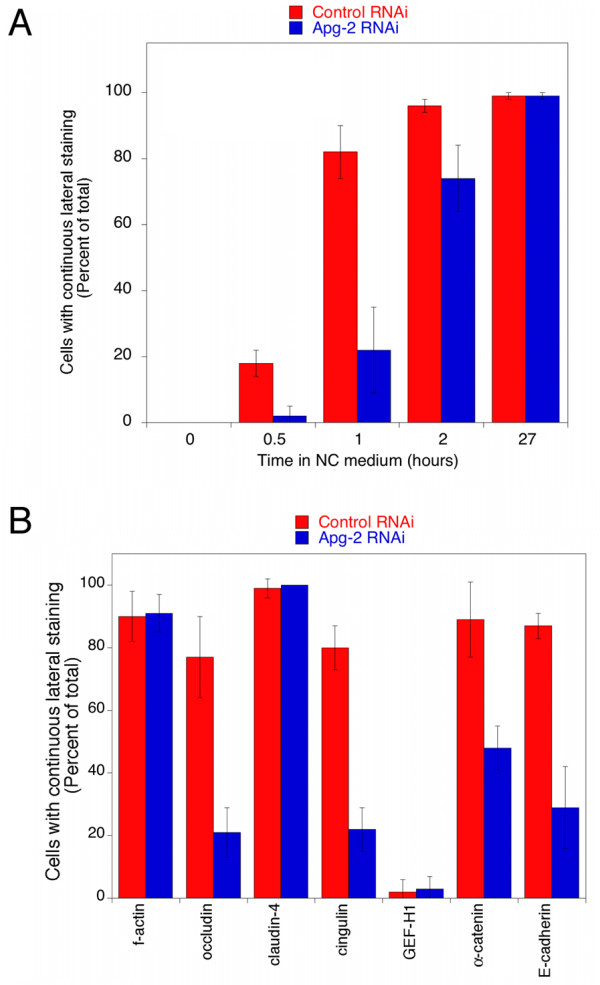
**Quantification of junction formation**. Control and Apg-2 RNAi cells were cultured and processed as described in figure 3. Junction formation was allowed to proceed for the indicated amount of time (A) or for one hour (B). The cells were then stained for either ZO-1 (A) or f-actin, occludin, claudin-4, cingulin, GEF-H1, α-catenin or E-cadherin (B). The fractions of cells expressing the labelled markers along the entire lateral cell membrane were then determined by counting. At least five different fields derived from two independent experiments were counted for each marker and time point. Note, as a large fraction of claudin-4 is continuously at the plasma membrane even before the addition of calcium, its early presence at the plasma membrane does not indicate tight junction formation.

We next analysed the distribution of ZO-1. In control cells, ZO-1 was quickly recruited to the forming junctional complex and formed complete junctional rings within 1 hour (Fig. 3B). In contrast, Apg-2 depletion inhibited junctional recruitment of ZO-1, and junctional rings were only complete after 2 hours (Fig. 3B and 4A). This inhibition is unlikely to reflect changes in the organisation of the actin cytoskeleton, as we could not detect obvious differences in the formation of cortical actin belts between control and Apg-2 depleted cells (Fig. 3C and 4B). However, it cannot be excluded that reduced Apg-2 expression caused more subtle changes in actin organisation. Thus, Apg-2 regulates the junctional recruitment of ZO-1 without strongly affecting the actin cytoskeleton.

**Figure 5:**
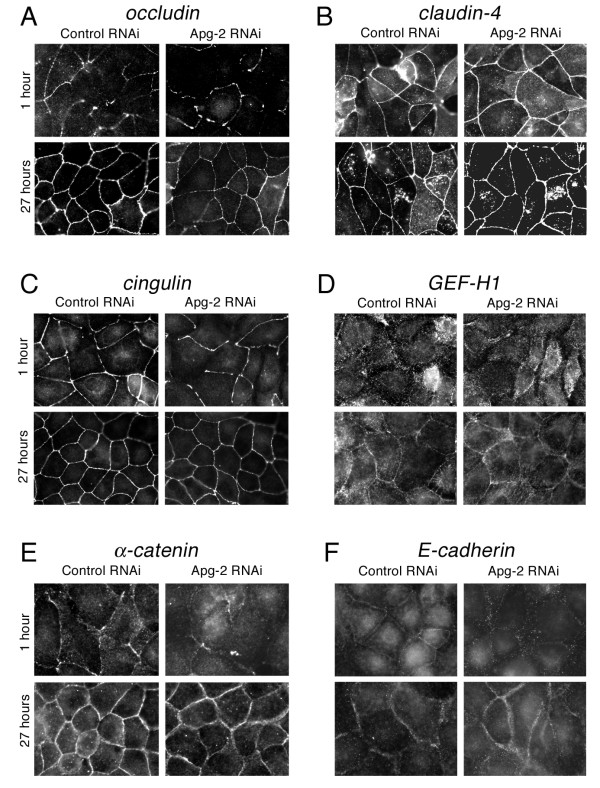
**Retardation of junction formation by Apg-2 depletion**. Control and Apg-2 RNAi cell lines were cultured and processed as in figure 3 and then stained with antibodies against the transmembrane proteins occludin (A) and claudin-4 (B), the junctional plaque components cingulin (C) and GEF-H1 (D), and the adherens junction proteins α-catenin (E) and E-cadherin (F). Shown are images derived from samples fixed after 1 hour and 27 hours of junction formation. Note, retardation of junctional recruitment was observed for occludin, cingulin, α-catenin and E-cadherin. GEF-H1 became only concentrated at tight junctions after longer time points of junction formation. Claudin-4 was present at the plasma membrane at all time points but only appeared to be concentrated at junctions at later time points. GEF-H1 and claudin-4 distributions were not affected by depletion of Apg-2.

We next determined effects on the recruitment of occludin and claudin-4, two transmembrane components of tight junctions [4,33]. Figure 5A shows that occludin concentrated more slowly at the forming junctional complex in Apg-2 depleted cells. Similarly, ZO-2 and ZO-3 were also recruited more slowly (not shown). Claudin-4, however, was more broadly distributed over the plasma membrane at early time points and there were no significant differences between control and depleted cell (Fig. 4B and 5B). Cingulin, a protein known to interact with ZO-1 [34], was also recruited more slowly to the forming junctions (Fig. 4B and 5C) whereas GEF-H1, which interacts with cingulin [35], was recruited only later to tight junctions even in control cells, and no differences were observed in Apg-2 depleted cells (Fig. 4B and 5D).

ZO-1 depletion has also been associated with a retardation in the maturation of adherens junctions from spot-like to belt-like junctions [36]. Hence, we tested whether Apg-2-depletion influenced the junctional recruitment of the adherens junction markers α-catenin, which is known to interact with ZO-1 [37,38], and E-cadherin. Indeed, α-catenin was recruited more slowly and E-cadherin required more time to form belt-like junctions (Fig. 4B, 5E and 5F). Thus, Apg-2 depletion retards tight junction formation as well as maturation of adherens junctions.

At later time points, the junctional distribution of tight and adherens junction markers was the same in Apg-2 depleted and non depleted cells. We could also not detect any differences in the Triton X-100 insolubility of different junctional proteins (not shown). Together with the functional data in figure 2, this suggests that Apg-2 is not required for junction formation but functions as a regulator.

### Apg-2 is required for epithelial morphogenesis in 3-D cultures

Apg-2 regulates signalling by ZO-1 and ZONAB, and both proteins have been associated with the regulation of epithelial morphogenesis of MDCK cells cultured in extracellular matrix gels [18,21]. Hence, we next tested whether Apg-2 depletion affected the capability of MDCK cells to form polarised hollow cysts in 3-D cultures.

**Figure 6:**
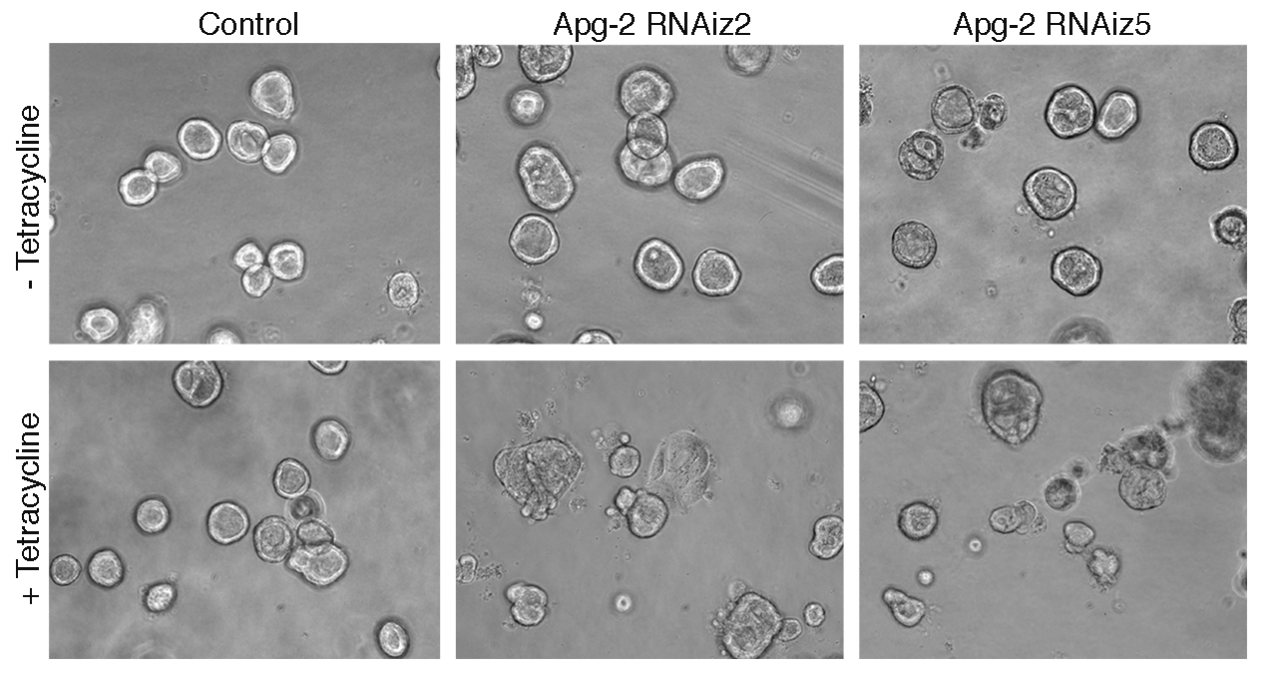
**Apg-2 depletion inhibits the formation of regular cysts in 3-D cultures**. Control and Apg-2 RNAi cell lines were cultured without or with tetracycline in collagen/matrigel gels. The cells were regularly inspected by phase contrast microscopy to monitor cyst and lumen formation. The shown images were taken 7 days after the cultures had been started. Note, most cysts formed by Apg-2 depleted cells had an irregular shape and lacked a lumen.

**Figure 7:**
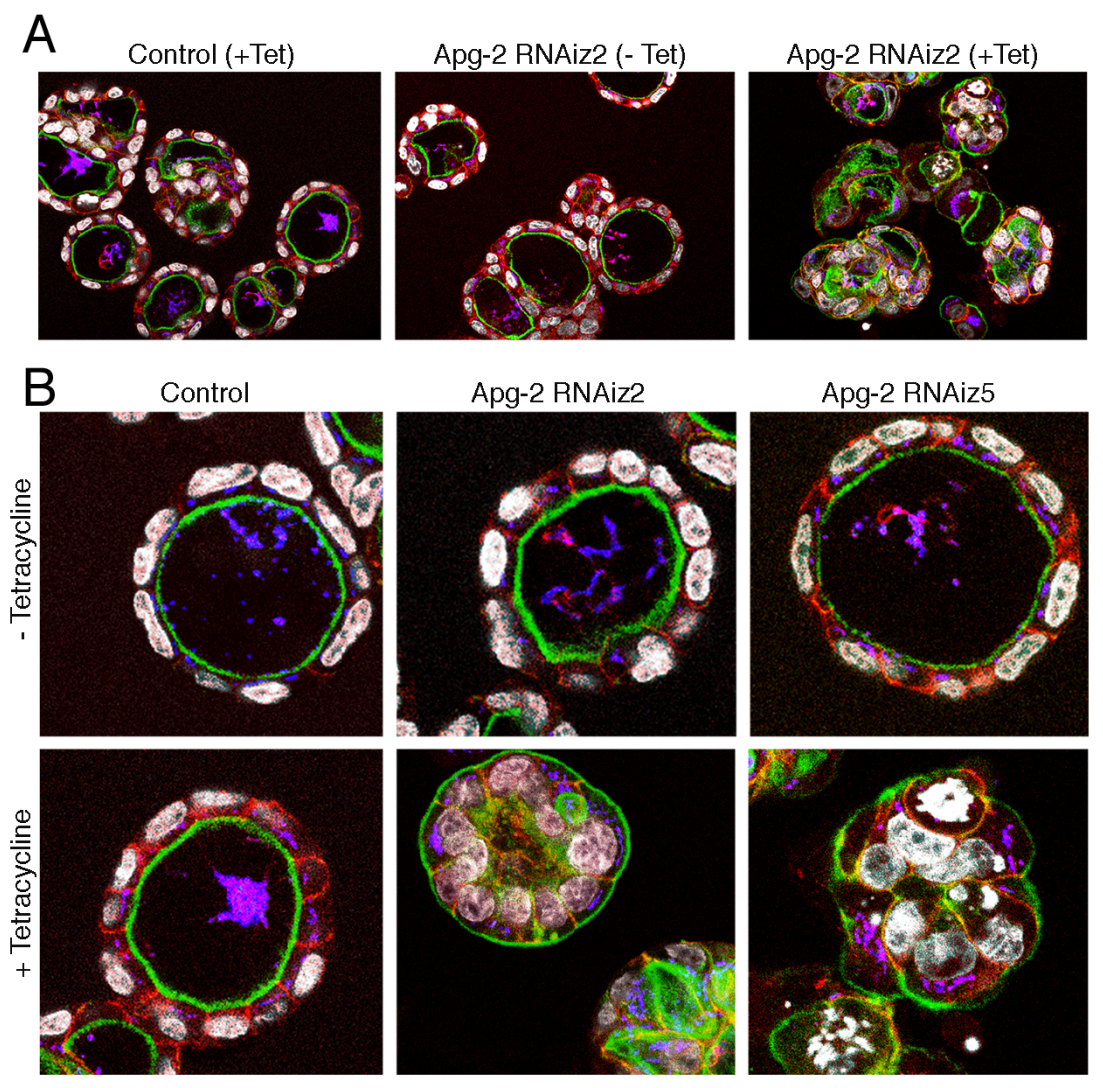
**Apg-2 depletion inhibits epithelial morphogenesis in 3-D cultures**. Control and Apg-2 RNAi cell lines were cultured as in figure 6. After fixation, the samples were labelled with FITC-phalloidin and antibodies against β-catenin (shown in red), the Golgi marker GP73 (shown in blue), and for DNA (shown in white). Panel B shows confocal sections taken at a higher magnification than those in panel A. Note, cysts formed by Apg-2 depleted cells were often not normally polarised and had irregular shapes.

**Figure 8:**
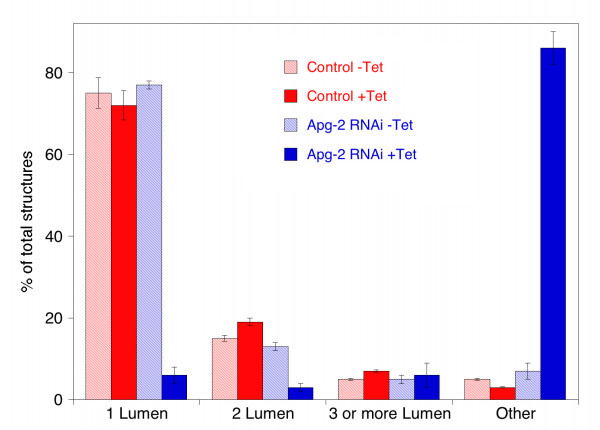
**Quantification of 3-D morphogenesis assays**. Control and Apg-2 RNAi cell lines were cultured and processed as in figure 6. In two experiments performed with different batches of matrigel, pictures from ten different areas were taken and quantified by counting the number and types of cultures. The formed structures were divided into the following classes: spherical structures with a single lumen; 2 lumen and 3 (or more lumen) but with a normal polarised organisation; and all other structures including cysts with no lumen.

Control cells and Apg-2 RNAi cell lines were cultured without or with tetracycline for 7 days and were then analyzed by phase contrast microscopy (Fig. 6) and immunofluorescence (Fig. 7). Tetracycline did not affect the capability of control cells to form polarised cysts as previously reported [18]. Apg-2 depleted cells, however, did not form regular structures and most of the cysts did not contain an obvious lumen. This was confirmed by counting the number of cysts formed by the different cultures: more than 70% of cysts formed by control cells or by Apg-2 RNAi cell lines in the absence of tetracycline formed cysts with a single lumen, but less than 10% did so when Apg-2 was depleted (Fig. 8).

**Figure 9:**
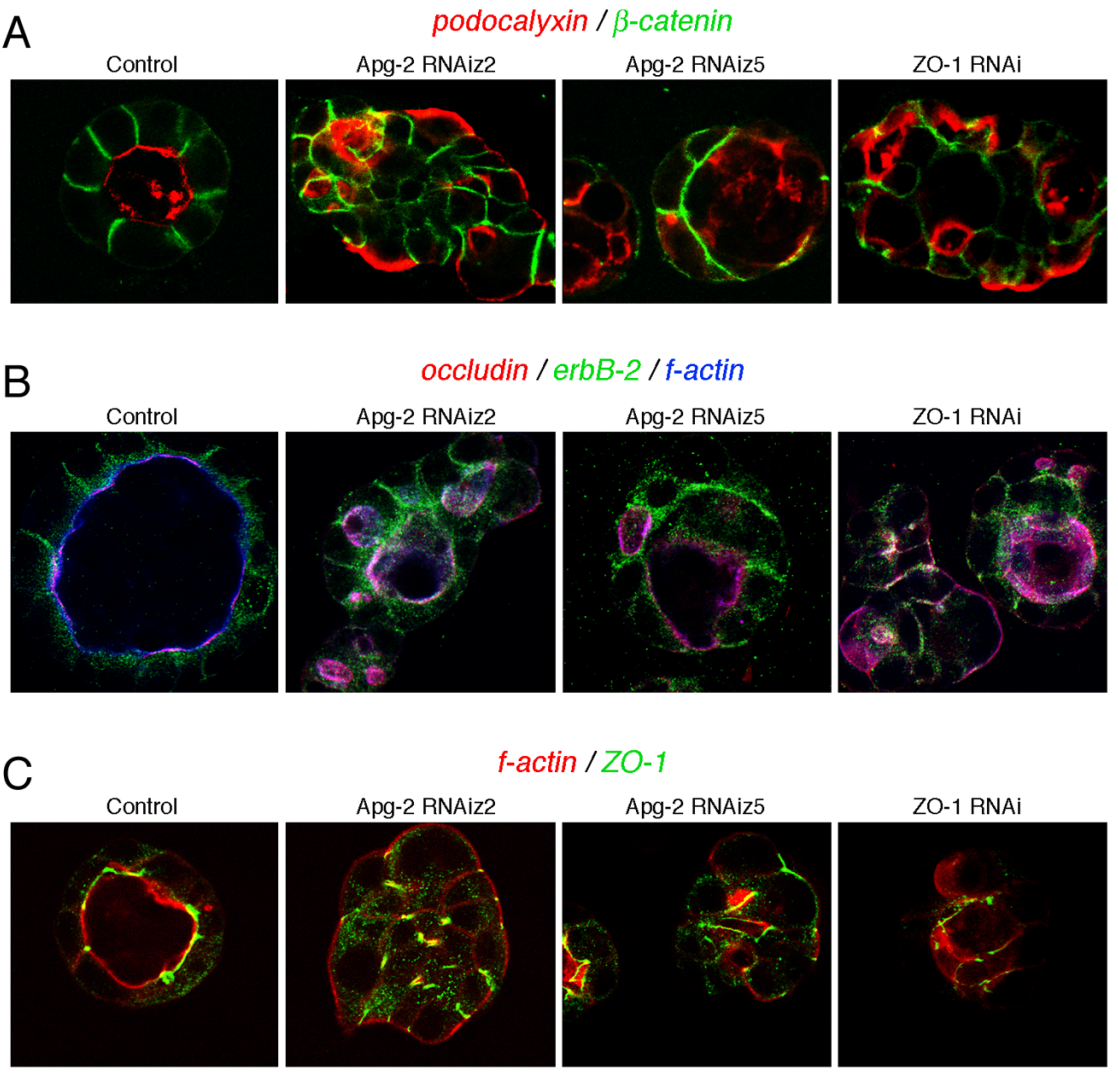
**Effect of Apg-2 and ZO-1 depletion on the organisation of 3-D cultures**. Control, and Apg-2 and ZO-1 RNAi cell lines were cultured as in figure 6. After fixation, the samples were labelled with antibodies against β-catenin and podocalyxin/GP135 (A); occludin, erbB-2 and with Alexa654-phalloidin (B); or with TRITC-phalloidin and anti-ZO-1 antibodies (C). Note, erbB-2 does not only localise to the lateral plasma membrane but also to cytoplasmic organelles, causing the cytoplasmic staining between the nucleus in the apical membrane in control cultures (B). Residual ZO-1 staining in ZO-1 RNAi cells was detected at cell-cell contacts (C).

Fluorescence microscopy revealed cysts formed by Apg-2 depleted cells did not achieve the normal polarised organisation. Whereas control cells formed a strong actin ring along the internal lumen, marking the apical pole of the cells; Apg-2 depleted cells had the strongest actin labelling along the external surface of cysts or in irregular internal structures (Fig. 7). Similarly, the Golgi apparatus was situated between the nuclei and the lumen in the control cells; in Apg-2 depleted cells, however, the Golgi apparatus was often detected between the nuclei and the external surface of the cysts (Fig. 7). Immunofluorescence experiments further showed that neither the apical marker podocalyxin nor not the basolateral plasma membrane protein erbB-2 achieved their normal polarised expression in Apg-2 as well as ZO-1 depleted cells (Fig. 9A and 9B). Staining for junctional markers such as occludin, ZO-1 and β-catenin also suggested that cells with reduced expression of Apg-2 or ZO-1 failed to form well-organised polarised cysts (Fig. 9). Thus, Apg-2 is required for the formation of polarised hollow epithelial cysts in 3-D cultures.

## Discussion

Tight junctions are composed of multimeric protein complexes often formed by different types of proteins that interact with each other. Epithelial junction formation is a sequential process that starts with the formation of a primordial adhesive junction that then matures into distinct tight and adherens junctions. ZO-1 can interact with different components of tight and adherens junctions and appears to play a modulatory role during formation of both types of junctions [16,17,36]. ZO-1 also regulates gene expression, cell proliferation and epithelial morphogenesis [18-20]. ZO-1's role in gene expression is regulated by an interaction with the heat shock protein Apg-2 [21]. Here we show that Apg-2 also modulates junction formation and is required for normal epithelial morphogenesis in 3-D cultures.

Depletion of Apg-2 affects junction formation in a similar way as depletion of ZO-1: formation of functional junctions was retarded, but not prevented, and the inhibition was only modest. In the case of ZO-1, it seems that its function in tight junction formation is at least in part redundant as depletion of ZO-2 in ZO-1 knockout cells prevents tight junction formation [39]. Whether MDCK cells express other proteins with a similar function as Apg-2 is currently not known; however, it is conceivable that other heat shock proteins might also aid junction formation. Alternatively, it could be that Apg-2 functions as a catalyst; hence, its function is not required, but its presence accelerates junction formation.

The mechanism by which Apg-2 regulates junction assembly is not known. It could be that Apg-2 regulates formation of ZO-1 complexes. We have thus far not been able, however, to detect differences in proteins co-precipitating with ZO-1 if Apg-2 was depleted (KM and MSB, unpublished). Nevertheless, it is possible that this involves interactions that are not detergent resistant or other unknown proteins. We could also not detect clear differences in the Triton X-100 insolubility of junctional proteins between control and Apg-2 depleted cells, but there was only a small pool of insoluble proteins at early time points when junction formation was inhibited (not shown). As we have only observed kinetic differences in junction formation, minor quantitative changes in complex formation might be involved that would be difficult to assess experimentally.

The SH3 domain of ZO-1, which is the domain that interacts with Apg-2, is not only required but also sufficient to rescue junction formation in ZO-1 depleted MDCK cells [16,21]. However, the SH3 domain by itself remains in the cytosol and is not recruited to cell junctions [18,19], suggesting that it binds to a cellular factor that does not need to be recruited to junctions. One such factor is the transcription factor ZONAB; however, neither overexpression nor depletion of ZONAB affects tight junction formation (KM and MSB, unpublished) [16]. Hence, one possibility is that ZO-1 regulates a function of Apg-2 rather than the other way around. In fact, ZO-1 does not bind the peptide-binding domain of Apg-2 but to the N-terminal domain containing the ATPase. Hence, ZO-1 binding might affect yet to be discovered functional properties of Apg-2 such as, for example, release of another protein bound to the peptide-binding domain. It will therefore be important to analyse how ZO-1 binding affects the biochemical properties of Apg-2 and to search for Apg-2 substrates and other interacting proteins.

Apg-2 also regulates epithelial morphogenesis in 3-D cultures. Apg-2 depleted cells did not form hollow cysts but poorly organised structures with none or several small lumen. The disorganised nature of these structures was evident from different immunofluorescence labellings that showed that their cells did not possess a uniform polarity or, in the absence of a clear lumen, maintained apical markers at the outer surface of the cysts. Strikingly, we observed the same phenotype in cells depleted of ZO-1. As overexpression of ZONAB also caused a similar phenotype, we considered it as likely that the reduced expression of ZO-1 affected morphogenesis by stimulating ZONAB [18]. Apg-2 depletion, however, does not stimulate ZONAB but inhibits it, as the two proteins compete with each other for binding to the SH3 domain of ZO-1 [21]. However, it is possible that deregulation, and not just activation, of the ZO-1/ZONAB pathway causes defects in the development of polarised hollow cysts. Alternatively, ZO-1 might regulate a function of Apg-2 that is important for morphogenesis. Moreover, Apg-2 is expressed in different subcellular locations and, like other heat shock proteins, might bind many different proteins and, hence, regulate different types of processes. Nevertheless, the similarity of the phenotypes in junction formation and 3-D morphogenesis caused by depletion of Apg-2 and ZO-1 suggests that the two interacting proteins function in at least overlapping pathways during epithelial differentiation.

## Conclusion

Depletion of Apg-2 in MDCK cells retards junction assembly and inhibits epithelial morphogenesis in three-dimensional cultures, indicating that Apg-2 is a functionally relevant regulator of epithelial differentiation.

## Methods

### Cell lines and culture conditions

Stable MDCK cell lines for conditional, tetracycline-induced RNAi of Apg-2 and ZO-1, as well as control lines were previously described [18,21]. For Apg-2, cell lines expressing shRNAs targeting two different sequences were used. For 3-D morphogenesis assays, the cells were plated in a matrigel/collagen mix and cultured for 7 days as detailed elsewhere [18]. For calcium switch assays, the cells were trypsinised for 20 minutes, resuspended in normal DMEM with 10% fetal calf serum, and incubated at room temperature for 15 minutes. The cells were then pelleted by centrifugation, resuspended in low calcium medium (spinner culture medium, Sigma) with 10% dialysed fetal calf serum, 2 mM L-glutamine and 1 mM sodium pyruvate). The cells were centrifuged again and resuspended in low calcium medium at a concentration of 1.5 × 10^6 ^cells/ml. 0.5 ml of this suspension was then plated per tissue culture insert (12-well clusters, 0.4 μm pore size, Corning). 1.5 ml of low calcium medium was added to the outer chambers. After 18 hours, the low calcium medium was replaced by standard tissue culture medium (DMEM with 10% fetal calf serum) and TER measurements were started after an initial incubation of 30 minutes at 37°C. For induction of RNAi, tetracycline was added to the low calcium medium at time of plating.

### Functional analysis of tight junctions

TER was measured at 37°C with an EVOM (World Precision Instruments) using an AC square wave current (+/- 20 mA, 12.5 Hz) and stick electrodes [40]. TER values for individual cultures were calculated by subtracting the values measured for filters without cells. For paracellular diffusion measurements, 4 kD FITC-dextran and 70 kD-Rhodamine B dextran (Sigma) were dissolved in medium and added to the apical chambers (1 mg/ml each) and diffusion to the basolateral side of the two dextrans was then assayed simultaneously as described [40]. Fluorescence was determined with a FLUOstar Optima (BMG LABTECH) plate reader (FITC-Dextran: Exc: 485 nm and Em: 544 nm and/or Rhodamine B-Dextran: Exc: 520 nm and Em: 590 nm).

### Immunoblotting

At the end of calcium switch experiments and permeability studies, filters were excised from their holders and transferred into tubes containing 300 μl of SDS-PAGE sample buffer. After separation on 5–15% gradient gels and transfer to nitrocellulose, expression of specific proteins was analysed by immunoblotting using the ECL detection kit (Amersham) [41]. Rabbit polyclonal antibodies specific for Apg-2, ZO-1, ZO-2, ZO-3 and mouse monoclonal antibody against α-tubulin were previously described [21,40,42,43].

### Immunofluorescence

Cells were fixed with either methanol, or paraformaldehyde and then permeabilised with 0.3% Triton X-100 as described [18,19,41]. Incubations with primary antibodies, and FITC- and Cy3-conjugated secondary antibodies (Jackson Immunochemicals, Inc.) as well as mounting of samples were performed as previously described for 2-D cultures [41] and 3-D cultures [18], respectively. Antibodies against Apg-2, ZO-1, ZO-2, ZO-3, GEF-H1 and E-cadherin were previously described [21,40,42,44]. The Golgi marker GP73 was labelled with an affinity purified rabbit polyclonal antibody raised against a recombinant protein containing the entire luminal domain. The antibodies against the following antigens were obtained from commercial sources: occludin, claudin-4, and cingulin, Zymed, Inc.; α-catenin, Sigma; β-catenin and erbB-2, Santa Cruz Biotechnology, Inc. The mouse anti-GP135/podocalyxin antibody was kindly provided by Dr. G. Ojakian (SUNY Downstate Medical Center, Brooklyn, New York, USA) [45]. Phase contrast and epifluorescence images were obtained with a Leica DM1 RB microscope equipped with a 63×/1.4 oil immersion objective and a Hamamatsu ORCA285 camera. Confocal images were acquired with eiher a Leica LCS SP2 or a BioRadiance2000 microscope also using a 63× oil immersion objectives. The image acquisition software supplied by the manufacturers was used to collect the images. Image brightness and contrast were adjusted with Adobe Photoshop.

## Authors' contributions

All authors performed experiments, designed the project and contributed to the writing of the manuscript. All authors approved the text and figures.
